# Aerosolized Particle Reduction: A Novel Cadaveric Model and a Negative Airway Pressure Respirator (NAPR) System to Protect Health Care Workers From COVID-19

**DOI:** 10.1177/0194599820929275

**Published:** 2020-05-19

**Authors:** Tawfiq Khoury, Pascal Lavergne, Chandala Chitguppi, Mindy Rabinowitz, Gurston Nyquist, Marc Rosen, James Evans

**Affiliations:** 1 Department of Otolaryngology, Head & Neck Surgery, Thomas Jefferson University Hospital, Philadelphia, Pennsylvania, USA; 2 Department of Neurosurgery, Thomas Jefferson University Hospital, Philadelphia, Pennsylvania, USA

**Keywords:** COVID-19, mask, aerosol, rhinology, skull base

## Abstract

**Objectives:**

This study aimed to identify escape of small-particle aerosols from a variety of masks using simulated breathing conditions. This study also aimed to evaluate the efficacy of a negative-pressure environment around the face in preventing the escape of small aerosolized particles.

**Study Design:**

This study is an evaluation study with specific methodology described below.

**Setting:**

This study was performed in our institution’s fresh tissue laboratory.

**Subjects and Methods:**

A fixed cadaver head was placed in a controlled environment with a black background, and small-particle aerosols were created using joss incense sticks (mass-median aerosol diameter of 0.28 µ). Smoke was passed through the cadaver head, and images were taken with a high-resolution camera in a standardized manner. Digital image processing was used to calculate relative amounts of small-particle escape from a variety of masks, including a standard surgical mask, a modified Ambu mask, and our negative airway pressure respirator (NAPR).

**Results:**

Significant amounts of aerosolized particles escaped during the trials with no mask, a standard surgical mask, and the NAPR without suction. When suction was applied to the NAPR, creating a negative-pressure system, no particle escape was noted.

**Conclusion:**

We present a new and effective method for the study of small-particle aerosols as a step toward better understanding the spread of these particles and the transmission of coronavirus disease 2019. We also present the concept of an NAPR to better protect health care workers from aerosols generated from the upper and lower airways.

At the time of the writing this manuscript, we are in the midst of a global pandemic the scale of which has not been seen in over a century.^1^ The coronavirus disease 2019 (COVID-19) has, in a matter of weeks, changed the way we practice medicine and conduct our daily lives.^2^ Hospitals around the nation are putting elective surgical procedures on hold to preserve personal protective equipment (PPE), keep ventilators available, and reduce the risk of transmission of COVID-19 to both patients and health care workers.^3^ Protecting health care workers while still performing the procedures our patients require has never been more important. In light of this, several studies and articles have recently been published attempting to quantify the amount of droplet and particulate matter generated by speech and sinonasal surgical procedures.^4,5^

There have been some valid concerns that the models published thus far do not evaluate the smallest aerosolized particles capable of transmitting COVID-19. The World Health Organization (WHO) has recognized that while standard transmission of COVID-19 is through droplets that are on the order of 5 to 20 µ, under some circumstances, the virus can be transmitted through an airborne mechanism, especially if procedures are being performed on the airway.^6^ This article serves as the first description of a method to visualize particles smaller than 5 µ and shows the utility of the testing method in evaluating a novel negative-pressure mask system that may help protect health care providers in a variety of situations.

## Materials and Methods

Institutional review board approval was obtained from the Thomas Jefferson University institutional review board. To generate a visible small-particle aerosol, joss incense sticks were used. The smoke generated from this type of incense has been shown to have a mass-median aerosol diameter of 0.28 µ and is white colored.^7^ Incense was burned in a collection vessel until it was filled with smoke. Smoke was then siphoned from a valve at the top of the collection vessel using a modified Ambu bag, and the process was repeated for each trial.

**Figure 1.**
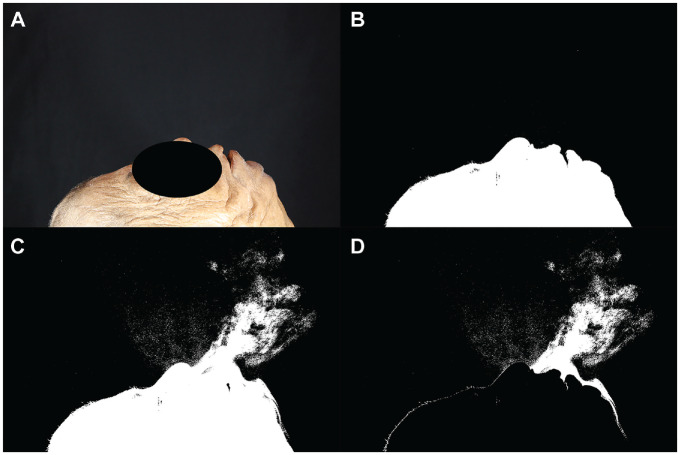
Trial without mask. (A) The first photo in the run without thresholding. (B) The first photo in the run after thresholding was performed. (C) This is the image 2 seconds into the run. A considerable amount of smoke can be seen emanating from the cadaver’s nose, mouth, and over the cadaver’s chin. (D) This image was generated by subtracting image B from image C.

**Figure 2.**
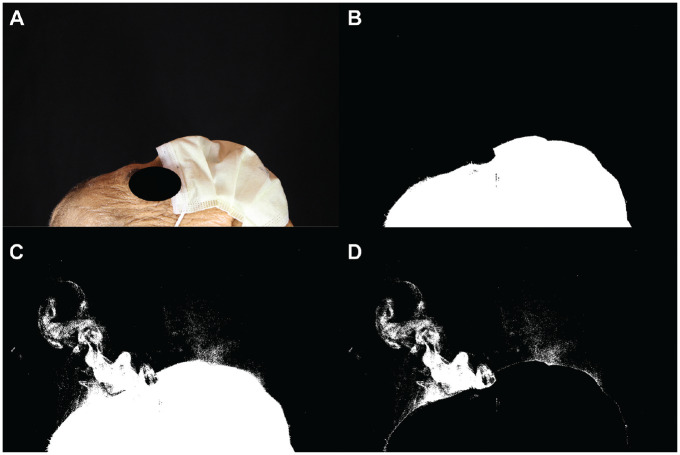
Standard surgical mask trial. (A) The first photo in the run without thresholding. (B) The first photo in the run after thresholding was performed. (C) This is the image 2 seconds into the run. Smoke was noted to escape from the top and sides of the mask. (D) This image was generated by subtracting image B from image C.

**Figure 3.**
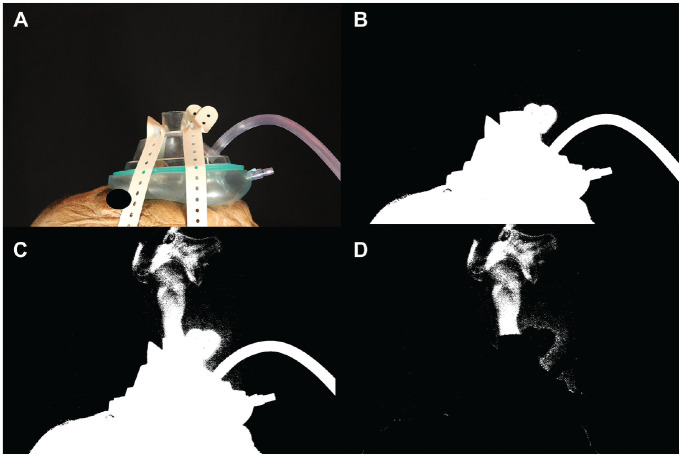
Negative airway pressure respirator (NAPR) with no suction. (A) The first photo in the run without thresholding. (B) The first photo in the run after thresholding was performed. (C) This is the image 2 seconds into the run. Smoke escaped from the aperture in the middle of the mask. (D) This image was generated by subtracting image B from image C.

**Figure 4.**
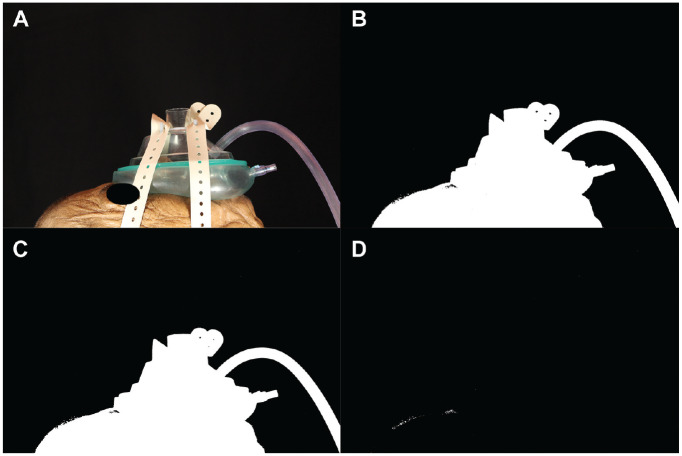
Trial with the negative airway pressure respirator (NAPR) with the suction at −120 mm Hg. (A) The first photo in the run without thresholding. (B) The first photo in the run after thresholding was performed. (C) This is the image 2 seconds into the run. No smoke was able to escape. (D) This image was generated by subtracting image B from image C.

A fixed cadaver head was placed in a controlled environment with a black background. Care was taken to minimize any extraneous light contamination. A size 8.0 endotracheal tube was then inserted into the trachea from below and the cuff was inflated. The smoke-filled modified Ambu bag was attached to the endotracheal tube and the contents of the bag were emptied over 3 seconds. This method was used to test several different scenarios: a cadaver with no mask (**Figure 1**), with a standard surgical mask (**Figure 2**), with a modified Ambu anesthesia mask (**Figure 3**), and with our novel mask—an Ambu mask fitted with suction tubing attached to a HEPA filtration system—which we have named a negative airway pressure respirator or “NAPR” (**Figure 4**). A digital camera with 18-megapixel resolution was used to capture the smoke escape from the cadaver, generating 36 images over each 3-second run. Using Adobe Photoshop v.20 (Adobe, Inc), a thresholding filter was applied to the first image in each run down to the noise floor (**Figure 1B**). A thresholding filter was then applied at the same level to the photo two-thirds of the way through each run (**Figure 1C**). Subtraction images were then generated by subtracting the processed first picture of each run to the processed picture two-thirds of the way through the run. A white pixel count was then performed to attempt to quantify the amount of smoke present in the field.

The pictures generated by this method contained 1.8 × 10^7^ pixels. Given the size of the field of view, each pixel represented an area on the order of 1 × 10^−5^ cm^2^ or 1000 µ^2^. Using thresholding, an environment with carefully controlled light, a rapid frame rate, and a stable setup, we were able to generate subtraction images that could identify exposure value difference ratios down to 1.08, which correlates to a 0.8% light emittance difference at the camera’s detector. Assuming the particles block light, this translates to the ability to detect particles down to a size of approximately 8 µ^2^.

**Figure 5.**
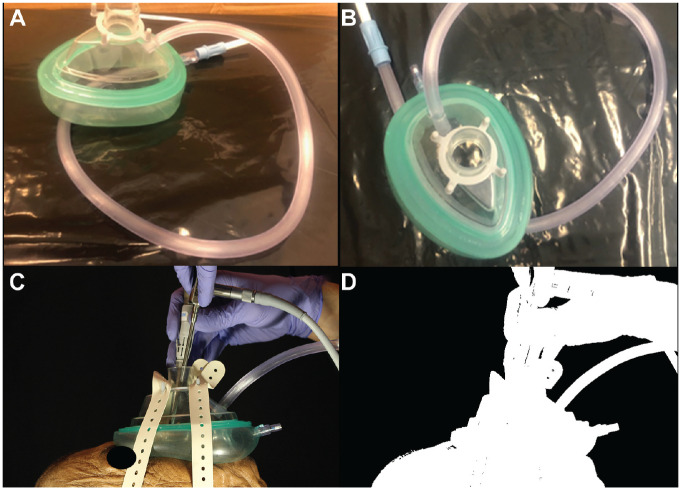
Working through the negative airway pressure respirator (NAPR). (A, B) These images show how a simple modification of an Ambu mask can create a negative-pressure environment to help protect health care workers when applied to a patient. (C, D) These images show working through the NAPR with no noted aerosol escape.

The specialized NAPR was designed by taking a standard Ambu mask, drilling a 9-mm hole in the plastic near the bottom of the mask, and inserting 10-mm diameter suction tubing through the new aperture (**Figure 5**). This mask was used to test the effect of a negative-pressure environment on the spread of aerosols using a pressure of −120 mm Hg.

## Results

The first trial was performed without any mask on the cadaver. It was clear that without a mask, a large amount of aerosolized particles were released (**Figure 1**). After thresholding and subtracting the image two-thirds of the way through the run with the first image of the run, 27,486 white pixels were detected (**Figure 1D**). When a standard surgical mask was added, the number of white pixels detected with this method was reduced to 21,379, but there were still aerosolized particles being released mainly from the top and sides of the mask (**Figure 2**). A trial was performed using the NAPR without any suction attached, and 3835 white pixels were detected (**Figure 3**). A trial performed after applying suction to the NAPR revealed 88 white pixels, which was due to background noise from a small amount of movement between the first photo and the photo used for analysis (**Figure 4**). This tiny amount of noise was present in all of the runs but was calculated to be <1% of the overall pixel count in each analysis. Trials were also performed in which suction was initiated halfway through the run, and it was noted that once suction was initiated, no additional particles escaped and particles seemed to regress from beyond the mask back into the negative-pressure environment. The amount of regression and escape was difficult to quantify due to motion artifact from connecting the suction tubing midway through the run and not having a consistent benchmark image with which to compare the first image. Trials were also performed using a drill and endoscope in the nose; while no detectable particle escape was noted, it was somewhat difficult to quantify given the motion artifact on the photos from the endoscope and drilling (**Figure 5**).

## Discussion

Here we present a method to test for the escape of small aerosolized particles from a patient’s airway as well as a novel negative-pressure mask concept. Based on our model, the NAPR mask seems to be protective against aerosol spread even in the scenario in which a patient is forcefully exhaling while an airway procedure is being performed. There have been multiple recent reports describing the risks of endonasal procedures with regard to transmission of COVID-19.^8^ Several interesting models have been recently published. Workman et al^4^ published a method using atomized fluorescein introduced into the nasal cavity through a defect in the cribriform. This interesting and timely article was primarily aimed at detecting large particles on the floor after endonasal procedures. Our work complements this by evaluating small- and medium-sized particles in an environment close to the patient’s face. In Workman et al,^4^ a mask was also proposed that limited the spread of large, fluorescein-coated tissue particles. In our study, we show that even with a surgical mask, small aerosolized particles can still escape if a patient is exhaling. Clearly, this would not be the case if the patient was intubated for surgery unless there was a cuff leak or endotracheal tube migration. However, when performing procedures on the upper and lower airways on an awake patient or when generating turbulent airflow in the airways of an intubated patient, small-particle aerosols can potentially be generated, and these were not able to be examined by methodology in Workman et al.^4^ For these reasons, we believe our findings to be germane to their results.

Similar to Workman et al,^4^ Anfinrud et al^5^ showed the patterns of droplet particle spread during speech in a recently published letter with accompanying video. By using a laser sheet and an iPhone, they were able to show that a surgical mask is able to contain most large droplet particles during speech. This is a useful experiment and certainly is applicable to everyday community transmission of COVID-19. However, Meselson’s comments^9^ on the article mentioned that this model may not be able to adequately capture smaller droplets and particles such as are present with COVID-19. While we are still learning more about this virus and the different methods by which it can transmitted, the WHO has put out guidelines cautioning that although COVID-19 is generally assumed to be transmitted via droplets, it can become airborne in circumstances including airway manipulation, and smaller particles need to be considered infective.^6^ There are also now multiple independent reports postulating that COVID-19 can indeed be spread through an airborne route.^10,11^

It is our belief that a local negative-pressure environment around the patient’s nose and mouth will be instrumental in minimizing the risk associated with procedures of the upper and lower airways. Based on our model, the NAPR was extremely effective at eliminating escape of small aerosolized particles even with simulated forced exhalation. We were able to adequately work in the nose given the constraints of the mask, but it could easily be modified further to facilitate other oral, laryngeal, or endonasal procedures, and other mask modifications are forthcoming (**Figure 5**). Given the nature of the current pandemic, using this mask on a patient while performing bronchoscopy could be a timely and helpful innovation.

This study has some limitations. Movements of the setup during the 3-second exhalation created some noise, which could affect analyzing the images; analyzing a 2-dimensional picture of a 3-dimensional reality always has certain limitations; and it is somewhat difficult to get each run perfect so that this method becomes very accurate quantitatively. Despite some inherent drawbacks, however, we feel that this is an excellent first step in measuring small-particle escape. Our model also represents a useful and effective method to test protective equipment. We also feel that the concept of an NAPR, irrespective of design, could potentially have far-reaching applications in the medical field. Future directions include refining our image-gathering and image-processing techniques, testing additional mask prototypes, and trials in live subjects.

## Conclusions

We present a new and effective method for the study of small-particle aerosols as a step toward better understanding the spread of these particles and the transmission of COVID-19. We also present the concept of a novel NAPR to better protect health care workers.
